# Superior Mesenteric Artery Syndrome Associated with Rapid Weight Loss Attributed to Amphetamine Abuse

**DOI:** 10.1155/2015/817249

**Published:** 2015-08-13

**Authors:** Richard M. Fazio, On Chen, Wael Eldarawy

**Affiliations:** ^1^Department of Internal Medicine, Maimonides Medical Center, 4802 Tenth Avenue, Brooklyn, NY 11219, USA; ^2^Department of Cardiology, Maimonides Medical Center, Brooklyn, NY, USA; ^3^Department of Gastroenterology, NY Methodist Hospital, 506 Sixth Street, Brooklyn, NY 11215, USA

## Abstract

Superior mesenteric artery (SMA) syndrome arises from a reduction in the angle formed between the SMA and the aorta, thereby compressing the third portion of the duodenum. This phenomenon may be caused by a number of factors, one of which being acute weight loss. We report a case of a female patient presenting with abdominal pain and vomiting who developed superior mesenteric artery (SMA) syndrome as a result of rapid weight loss, thought to be secondary to amphetamine abuse. This association can often be overlooked and, to our knowledge, has not been previously reported.

## 1. Introduction

**Figure 1:**
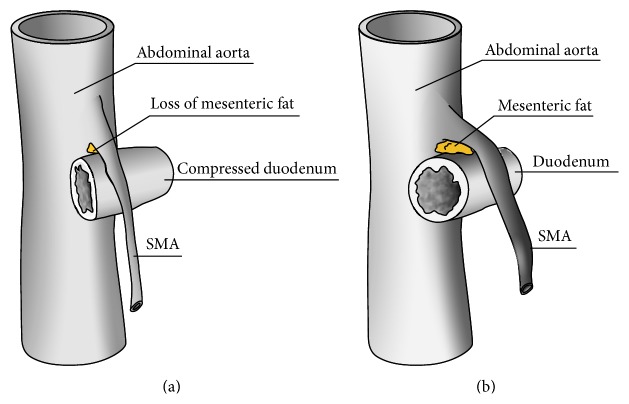
A schematic view of the aorta, SMA, and third portion of the duodenum. (a) Loss of mesenteric fat producing an acute angle between aorta and SMA and compression of the duodenum. (b) Presence of mesenteric fat and a normal angle between aorta and SMA.

The superior mesenteric artery (SMA) syndrome, also known as Cast Syndrome, Mesenteric Root Syndrome, or Wilke's disease, is a rare cause of mechanical small intestinal obstruction and classically associated with rapid weight loss. SMA syndrome stems from compression on the third portion of the duodenum, resulting from the acute angle created between the superior mesenteric artery and the aorta, as caused by paucity of mesenteric and retroperitoneal fat pads. An illustration of this concept can be seen in Figure 1.

## 2. Case Report

**Figure 2:**
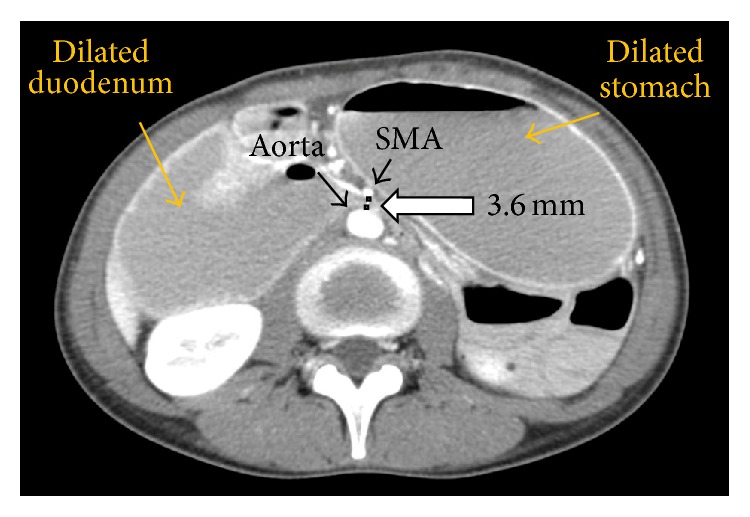
Contrast CT of the abdomen showing the distance between aorta and SMA (white arrow, marked as 3.6 mm). Also seen are a dilated stomach and duodenum and location of the SMA (black arrow).

**Figure 3:**
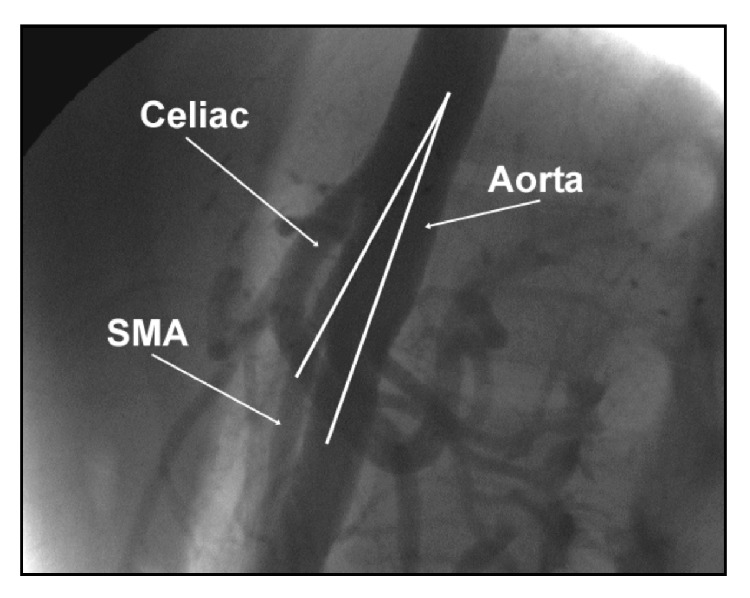
Angiography of the abdominal aorta. The celiac and SMA branches are identified, as well as a narrow-angle formed between the abdominal aorta and the SMA branch.

The patient being presented is a 30-year-old Asian-American female with a past medical history significant for Attention Deficit Hyperactivity Disorder (ADHD) and bipolar disorder and is prescribed a daily regimen of 10 mg amphetamine/dextroamphetamine. She presented with right upper quadrant and epigastric pain of 5 days' duration. The pain was described as sharp, constant, and exacerbated by eating. It was associated with bilious, nonbloody vomiting, nausea, anorexia, and a thirty-pound weight loss over a time period of two months. The patient stated that she was under a significant amount of stress and she recently increased the dosage of her amphetamine/dextroamphetamine, the exact amount of which she was not able to specify. Examination revealed cachexia (BMI of 15.6) and right upper quadrant tenderness upon palpation. Abdominal CT scan (Figure 2) revealed a markedly dilated stomach and duodenum and paucity of intraperitoneal fat. Mesenteric angiography (Figure 3) showed a 9-degree angle between the SMA and aorta (normal, 25–60 degrees) and an aortomesenteric distance of 3.6 mm. (normal, 10–28 mm) [1–4]. The patient was treated with nasogastric intubation for gastric decompression, total parenteral nutrition, and antiemetic medication to prevent further vomiting episodes. Incremental advancements of enteral feeds were introduced to prevent electrolyte changes seen in refeeding syndrome and the concept of positional feeding (i.e., having the patient in a prone or left lateral decubitus position during feeding) was strongly encouraged. Symptoms continuously improved through her hospitalization, and she was eventually discharged with the ability to tolerate oral feeding. The patient was seen by representatives of the psychiatric department while an inpatient and was scheduled for outpatient follow-up with eventual adjustment of home medications and discontinuation of amphetamines. Eight months later, she presented to the outpatient medical clinic having reported appropriate weight gain without further symptoms.

## 3. Discussion

Located in the retroperitoneum, the third portion of the duodenum runs through the acute angle created by the aorta and its branching artery known as the superior mesenteric artery [5, 6]. The normal aortomesenteric angle ranges from 25 to 65 degrees due to the retroperitoneal fat pads that act as a cushion, preventing the SMA from compressing the duodenum. The mean aortomesenteric distance, which is directly proportional to the amount of “cushion” available, measures approximately 10–28 mm in a healthy person [2–4, 6, 7]. Aortomesenteric angles less than 22–25 degrees and distances less than 8 mm have correlated well with the diagnosis of SMA syndrome [3, 7]. Predisposing factors include a prolonged supine position, an unusually low origin of the SMA, exaggerated lumbar lordosis, and an abnormally high fixation of the duodenojejunal flexure to the ligament of Treitz. Precipitating factors are those that cause marked weight loss resulting in loss of the retroperitoneal fat pad, such as cancer and burns, anorexia nervosa, IV drug abuse, malabsorption, postoperative state disease, trauma to the spine, and application of a body cast [5, 6, 8, 9]. Symptoms of SMA syndrome are those of duodenal compression, namely, nausea, vomiting, eructation, abdominal pain, early satiety, distension, tenderness, and abnormal bowel sounds [10–12]. They are worsened in the postprandial period and may be alleviated by maneuvers which increase the angle between the SMA and the aorta, including lying in either the prone or left lateral decubitus position and when performing the knee-to-chest maneuver [5, 13, 14]. This feature of postprandial abdominal pain may further compound the problem, as it will lead to continued weight loss and thus perpetual symptoms [14]. The diagnosis of SMA syndrome is oftentimes overlooked due to the broad spectrum of differentials associated with these generalized GI symptoms. Also, other conditions may be found in association with this disease, the most common of which being peptic ulcer disease. Hyperchlorhydria is formed in response to the duodenal distention and gastric stasis, which may precipitate the formation of ulcers, along with progressive esophagitis and gastritis [7, 15]. Classically, SMA syndrome is diagnosed via upper GI series or CT imaging with oral contrast, which will illustrate proximal duodenal dilatation, obstruction of the third part of the duodenum, and delay of barium passage into the distal duodenum; however, more modern modalities include SMA angiography to measure the angle between the aorta and the SMA, as well as esophagogastroduodenoscopy to demonstrate compression of the duodenum [1, 2, 5, 13, 14, 16]. Treatment of SMA syndrome begins with a conservative approach, especially for those in the acute setting of the disease. This includes promotion of weight gain either by TPN or by enteric feeding, in order to restore the aortomesenteric angle, correction of fluid, and electrolyte balance, and addressing any underlying precipitating factors [2, 5, 10, 14]. Surgical intervention becomes necessary when there is failure following conservative medical therapy and progressive weight loss in the setting of longstanding disease or as a result of complicated peptic ulcer disease [9, 17]. The goal of a surgical approach is duodenal decompression, which may be achieved through bypass of the obstructed segment of small bowel, as seen in both gastrojejunostomy and duodenojejunostomy techniques or through mobilization of the duodenum, as performed during Strong's procedure. Duodenojejunostomy has become the preferred procedure amongst the surgical community due to its higher success rates and decreased risk for bacterial overgrowth syndrome. In particular, a laparoscopic approach has drawn favor over the traditional open techniques as a way to decrease morbidity, shorten hospital stays, and increase the overall success rate of the procedure [14, 18].

## 4. Conclusion

The FDA approves the use of amphetamines for indications such as ADHD and narcolepsy; however, the off-label use and abuse of these drugs have grown to become a dangerous epidemic in the United States in recent years, most notably amongst young, college-aged individuals. Amphetamine abuse may involve acquisition from illicit sources or manipulation of legal doses, which we suspect led to our patient's rapid weight loss and SMA syndrome. Amphetamine abuse may be difficult to recognize, and a patient's history is oftentimes withheld. Therefore, when presented with a patient complaining of abdominal symptoms mimicking small bowel obstruction in the setting of rapid weight loss, one should consider the diagnosis of amphetamine-induced weight loss causing SMA syndrome.
